# Good outcome following liver transplantation using pericardial-peritoneum window for hepato-atrial anastomosis to overcome advanced hepatic alveolar echinococcosis and secondary Budd-Chiari Syndrome - a case report

**DOI:** 10.1186/s12893-017-0205-2

**Published:** 2017-01-13

**Authors:** Konrad Kobryń, Rafał Paluszkiewicz, Krzysztof Dudek, Urszula Ołdakowska-Jedynak, Michał Korba, Joanna Raszeja-Wyszomirska, Piotr Remiszewski, Michał Grąt, Piotr Milkiewicz, Waldemar Patkowski, Marek Krawczyk

**Affiliations:** 1 Department of General, Transplant and Liver Surgery, Medical University of Warsaw, Poland. Banacha Street 1a, 02-097 Warsaw, Poland; 2 Department of Hepatology and Internal Diseases, Medical University of Warsaw, Warsaw, Poland

**Keywords:** Atrium–IVC anastomosis, Budd-Chiari Syndrome, Hepatic alveolar Echinococcosis, Liver transplantation, Pericardial-peritoneum window, Case report

## Abstract

**Background:**

This report presents a case of a 57- year old female with advanced Hepatic Alveolar Echinococcosis causing a secondary Budd-Chiari Syndrome due to infiltration of the suprahepatic inferior vena cava treated successfully by liver transplantation.

**Case presentation:**

A temporary veno-venous bypass was introduced, but a typical end to end cavo-caval anastomosis wasn’t possible in this case. In order to access a disease free part of the inferior vena cava, an oval window of the diaphragm was excised, providing communication between the peritoneum and pericardium. A vascular clamp was placed onto the right atrium which allowed for an atrial-caval anastomosis. The remainder of hepatectomy was performed in a conventional manner. In the post-operative period and during the 18 month follow-up there were no complications. The patient remains in good general condition with optimal graft function.

**Conclusions:**

A hepato-atrial anastomosis with a pericardial-peritoneum window during liver transplantation is feasible and extends the curability potential for patients with advanced Hepatic Alveolar Echinococcosis considered for liver transplantation.

## Background

Advanced hepatic alveococcosis due to its volume may interfere with the hepatic veins and inferior vena cava (IVC) causing Budd-Chiari syndrome (BCS) which is an indication for liver transplantation (LTx). The thrombosis and stricture may be found at any point, ranging from the hepatic veins to the right atrium along the IVC [1, 2]. When this is the case, hepatectomy of the diseased liver requires supra hepatic IVC clamping at the right atrium, with later end-to-end anastomosis with the donor liver IVC cuff [3–5]. Our report presents a rare approach during hepatectomy, perioperative management and excellent postoperative results of the patient.

## Case presentation

A 57 year old female patient was qualified for LTx due to BCS caused by an advanced *Echinococcus multilocularis* infection. The patient received Albendazole treatment for several years which was terminated two years prior to transplantation due to a developed post-drug pancytopenia. No other anthelminthic drugs were administered until LTx. Furthermore, portal hypertension, esophageal and gastric varices, portal hypertensive gastropathy, and ascites were present at time of transplant qualification. Celiac disease, gastro-esophageal reflux disease (GERD), and L1 vertebral fracture were associating commorbidities.

**Fig. 1:**
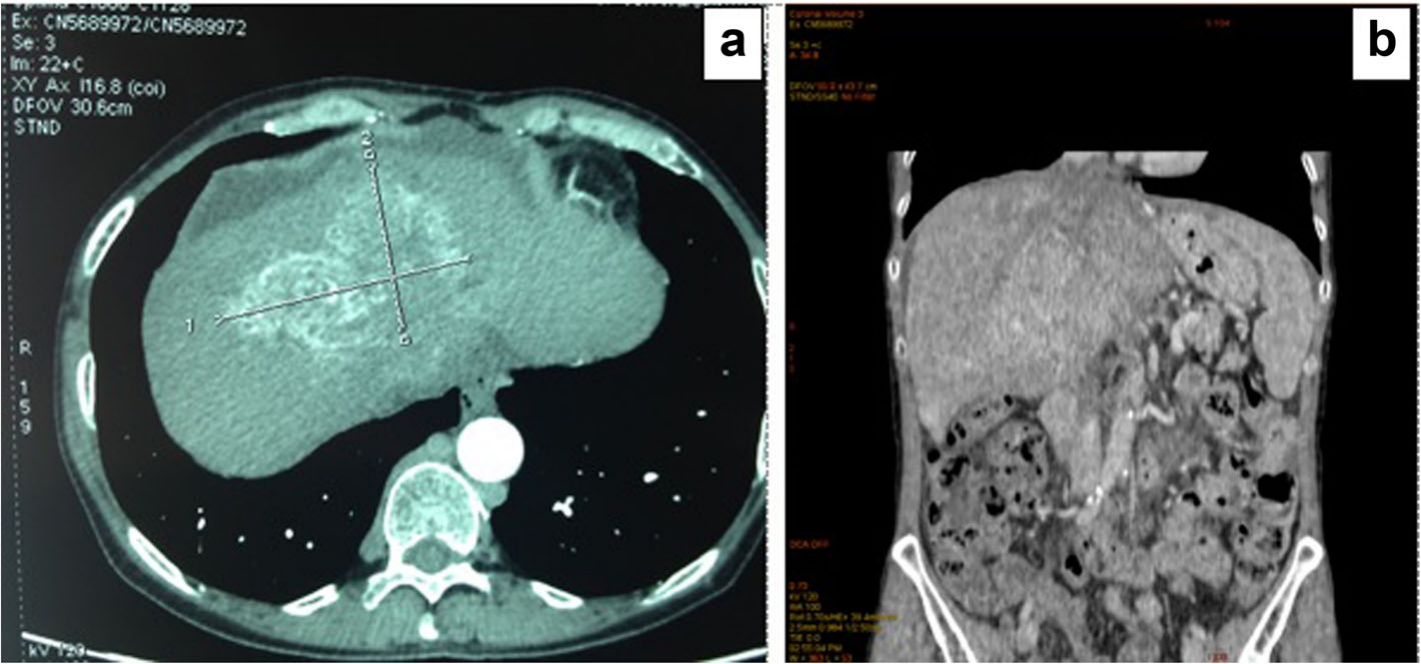
Pre-Transplant CT-scans presenting: **a** Computed Tomography of a 110 × 75 × 70 mm hepatic alveolar echinoccocal cyst located in segments III–VIII; **b** Hepatic vein thrombosis – Budd-Chiari Syndrome

The size of the alveolar lesion in segments III–VIII was 110 × 75 × 70 mm (Fig. 1a) and involved the hepatic vein confluence, causing narrowing of the IVC lumen with mild but disturbed blood flow. The intrahepatic IVC was free of abnormalities. The lesion occluded the left and middle hepatic veins, extending towards the confluence of the right hepatic vein with the IVC (Fig. 1b). The lumen was narrowed, with an organized thrombus visible on ultrasound, but blood flow was maintained.

**Fig. 2:**
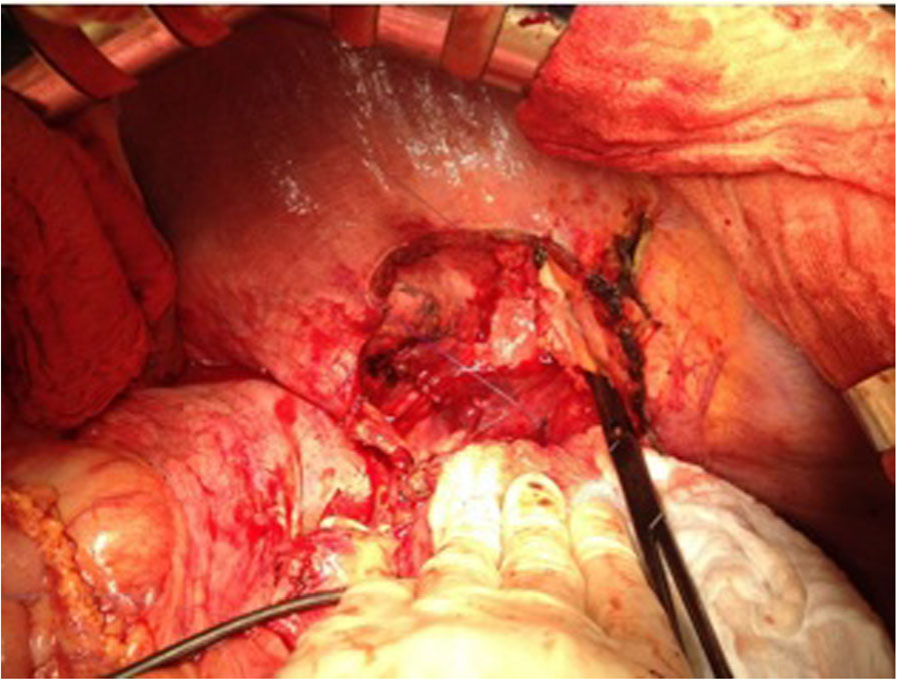
Clamp applied to the right atrium and positioning of the sutures

**Fig. 3:**
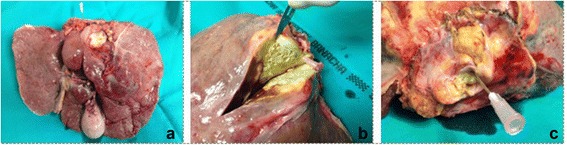
**a** Explanted diseased liver; **b** Alveolar Cyst with *E. multilocularis* in the explanted liver; **c** Strictured lumen of the supra hepatic inferior vena cava, which was invaded by an alveolar lesion (marked by needle). Dissected diaphragm measured 10 × 10 cm

The donor liver was retrieved from a 43 year old dead brain donor (DBD) matching the recipient’s blood type and similar weight and height values. The LTx began on the 16^th^ September, 2014 with the use of an extra-corporeal veno-venous bypass. The portal vein and hepatoduodenal ligament was difficult to dissect as it was embodied in post-inflammatory scar tissue. The suprahepatic IVC along with the surrounding diaphragm was involved in the alveolar lesion. A 10 × 10 cm window of the diaphragm was excised creating an opening of the right pleural cavity and pericardium (Fig. 2). Thus, with the exposure of the pericardial part of the IVC, the vascular clamp was placed on the atrium because the whole IVC was infiltrated by the lesion. The infrahepatic part of the IVC was clamped in a standard procedure. Both ends of the IVC were then cut, and the liver was explanted along with its intrahepatic IVC (Fig. 3a). The clamping of the right atrium caused cardiac arrhythmia and the electrocardiogram showed signs of myocardial ischemia. This was managed by the anesthesiologist and hemodynamic stability was achieved throughout the operation.

Two 24 fr pleural drains were placed into the apex and base of the right pleural space. The supra hepatic IVC of the donor graft was sutured directly to the right atrium using an end-to-end anastomosis. The excised window in the diaphragm was minimized by suturing its free ends together without the use of a Prolene patch, and without complete closure of the diaphragm in order to avoid stenosis of the IVC–atrium anastomosis. Communication between the pericardium and right pleural cavity was intentionally sustained to allow for fluid drainage. After completion of the portal anastomosis clamps from the IVC and portal vein were removed. A mild, 1-min reperfusion syndrome with a systolic pressure of 60 mmHg was observed after 8 h 6 min of cold ischemia. At that point, pleural drainage was enabled. Further steps during transplantation were performed in a standard procedure. Intraoperative blood loss was estimated at 1000 ml; thus, 2 units of red blood concentrate and 4 units of fresh frozen plasma were transfused. The anhepatic phase was 136 min. Total operation time was 6.5 h.

**Fig. 4:**
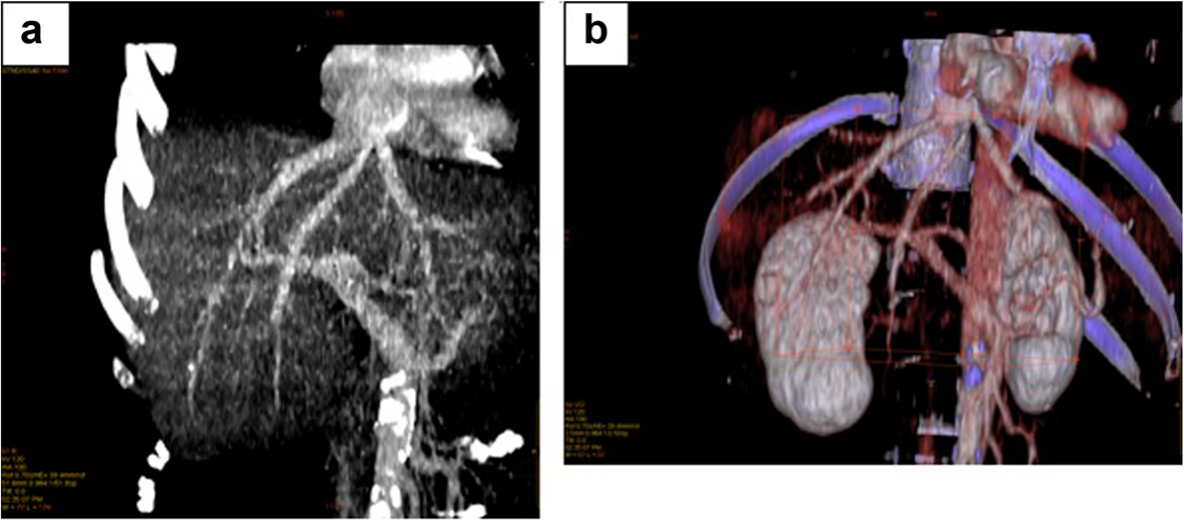
Post-transplant computed tomography scans: **a** Anterior CT image of hepato-atrial (atrial-caval) anastomosis; **b** 3-Dimensional computed tomography reconstruction of the vascular image presenting the hepato-atrial anastomosis

An abdominal ultrasound was performed on the first post-operative day (POD), showing good vascular flow. An echocardiography performed on the second POD found no abnormalities. The pleural drain was removed on the third POD and a chest X-ray confirmed no signs of pneumothorax or effusion in the right pleural space. Time in the intensive transplant care unit was 3.5 days. The immunosuppressive regimen consisted of tacrolimus 0.05 mg/kg × 2 and prednisolone 20 mg × 1. The patient’s liver tests recovered gradually. Computed tomography showed satisfactory and functioning hepato-atrial anastomosis (Fig. 4a and b). She was discharged on POD 25 and continued with tacrolimus 7 mg × 2, mycophenolate mofetil 500 mg × 2 and albendazole treatment 400 mg × once daily. Intolerance of the latter drug was not observed during hospitalization. At an 18-month follow-up and recurrence free period, the patient is doing well with good overall outcome and graft function.

## Discussion

Hepatic alveolar echinococcosis (HAE) is a parasitic disease that develops slowly. Very often, the first symptoms manifest, as in our case of *E. multilocularis* (Fig. 3b), at a very advanced, mature stage. In the first imaging examinations, HAE can resemble a tumor like hepatocellular carcinoma. Clinical signs, such as mechanical jaundice, repetitive cholangitis, liver abscess, portal hypertension, esophageal varices, and sepsis are secondary to the disease. Additionally, Budd-Chiari Syndrome might be present [3–6].

Surgical resection usually is the main treatment for this disease; however, because alveolar symptoms tend to be absent for a long period of time, the cysts tend to be very advanced at diagnosis. Large lesions without response to drug treatment and with invasion of the vascular tree (Fig. 3c) of the liver will eventually develop BCS, thus should be directly qualified for LTx. This seems to be the only final treatment for HAE, and 90% of patients die within 10 years without surgery [4–7].

LTx due to HAE has been reported to have increased morbidity and mortality. A thorough examination is compulsory with MRI and three-dimensional computed tomography reconstruction in order to minimize intraoperative risk [7]. An early remark on the use of veno-venous bypass allows for better intraoperative preparation and greatly minimizes the risk of bowel congestion, shortens the hepatectomy time and limits the need for excessive maneuvers with the diseased liver, reducing the risk of tissue impairment and excessive bleeding [8–12]. A pericardial-peritoneum window as a procedure has been reported many times, but is performed due to malignant diseases or pericardial effusion and not during LTx [8–10, 12–14].

Several authors state that a pericardial-peritoneum window acts as a collection chamber for pericardial fluid, which is later absorbed through the peritoneum [9, 10]. In our opinion, the implantation of the donor liver through such a suprahepatic atrium-IVC cuff end-to-end anastomosis allows both lobes of the liver to firmly press against the diaphragm. This creates a continuation of the thoracic-abdominal barrier, simultaneously avoiding future diaphragmatic hernia. Thus, leaving a 20-40-mm opening around the IVC anastomosis avoids its occlusion in the future, provides prompt drainage of fluid, and leaves the diaphragm under less tension. There has been several publications, mainly for initial BCS, presenting different solutions of cavoplasty in order to perform an atrial-caval anastomosis [15, 16]. Though principles are similar, our report presents how to manage a rare case of advanced HAE and in result secondary BCS.

## Conclusions

LTx is feasible when performing an IVC hepato-atrial anastomosis. Patient’s outcome is not impaired, and the post-operative results, follow up, and quality of life is good. Therefor even advanced cases of HAE may be successfully treated with liver transplantation.

## Abbreviations

BCS: Budd-Chiari Syndrome
CD: Celiac Disease
EV: Esophageal Varices
GERD: Gastro-Esophageal Reflux Disease
HAE: Hepatic Alveolar Echinococcosis
IVC: Inferior vena cava
LTx: Liver transplantation
PDPS: Post-drug pancytopenia syndrome
PHG: Portal Hypertensive Gastropathy
POD: Post-operative day
